# Accuracy of MicroRNA Discovery Pipelines in Non-Model Organisms Using Closely Related Species Genomes

**DOI:** 10.1371/journal.pone.0084747

**Published:** 2014-01-03

**Authors:** Kayvan Etebari, Sassan Asgari

**Affiliations:** School of Biological Sciences, The University of Queensland, Brisbane, Queensland, Australia; Swedish University of Agricultural Sciences, Sweden

## Abstract

Mapping small reads to genome reference is an essential and more common approach to identify microRNAs (miRNAs) in an organism. Using closely related species genomes as proxy references can facilitate miRNA expression studies in non-model species that their genomes are not available. However, the level of error this introduces is mostly unknown, as this is the result of evolutionary distance between the proxy reference and the species of interest. To evaluate the accuracy of miRNA discovery pipelines in non-model organisms, small RNA library data from a mosquito, *Aedes aegypti*, were mapped to three well annotated insect genomes as proxy references using miRanalyzer with two strict and loose mapping criteria. In addition, another web-based miRNA discovery pipeline (DSAP) was used as a control for program performance. Using miRanalyzer, more than 80% reduction was observed in the number of mapped reads using strict criterion when proxy genome references were used; however, only 20% reduction was recorded for mapped reads to other species known mature miRNA datasets. Except a few changes in ranking, mapping criteria did not make any significant differences in the profile of the most abundant miRNAs in *A. aegypti* when its original or a proxy genome was used as reference. However, more variation was observed in miRNA ranking profile when DSAP was used as analysing tool. Overall, the results also suggested that using a proxy reference did not change the most abundant miRNAs’ differential expression profiles when infected or non-infected libraries were compared. However, usage of a proxy reference could provide about 67% of the original outcome from more extremely up- or down-regulated miRNA profiles. Although using closely related species genome incurred some losses in the number of miRNAs, the most abundant miRNAs along with their differential expression profile would be acceptable based on the sensitivity level of each project.

## Introduction

microRNAs (miRNAs) are small non-coding RNAs of ∼22 nucleotides, which are highly conserved among evolutionarily related species, and many even have homologs in distantly related species [1]. They have added a new facet of control to the complex network of gene transcription pathways and regulate around 30–75% of different mRNA transcripts in eukaryote cells [2]. miRNAs regulate the expression of target genes by binding to complementary sequences in the target mRNA and play important roles in various biological processes through post-transcriptional regulation of gene expression. Differential expression of miRNAs under various biological conditions, such as development, immune challenge, host-microorganism interactions and stresses has been reported in many species [3]–[11]. These characteristics have made some miRNAs suitable biomarkers for disease diagnostics [12], [13].

In recent years, the number of miRNA annotations has increased particularly in large and/or poorly annotated genomes [14] due to the large amount of sequencing data that can readily be produced by next generation sequencing platforms, such as the Illumina and Solexa. Since identifying the first miRNA in *Caenorhabditis elegans* [15], massive numbers of miRNAs have been identified in other model organisms. In the last few years, by increasing our knowledge of miRNA biology and also significant reductions in sequencing costs, the number of research projects with a focus on the role of miRNA under different biological conditions in non-model organisms has also increased. Due to improvements in prediction algorithms, miRNA discovery from various non-model organisms has advanced, with 21,264 miRNAs known to date (miRBase v19.0). There are a few technical factors such as sequencing accuracy, genomic mapping efficacy, and small RNA library preparations, which make small RNA-seq (smRNA-seq) data interpretation a daunting task [14], [16]. Using this technology in a species lacking genomic resources is quite challenging due to high levels of small RNA diversity and concerns over read mapping accuracy in the absence of a genome scaffold.

In many studies, detection of known/conserved miRNAs and their expression levels is a priority rather than discovery of novel miRNAs. In this case, mapping millions of sequencing reads to a reference genome can be replaced by aligning these small reads against the sequences of known miRNAs in other species. Many miRNA analysis tools, such as miRExpress [17] and DSAP [18], have been developed based on this approach which could be used to determine miRNA expression profiles when genomic sequences are unavailable. Using other organisms’ genomes as proxy reference to identify conserved miRNAs in non-model species has been approached by many research groups [19]–[22]. How well do miRNA discovery pipelines perform for miRNA discovery analysis from smRNA-Seq data in the absence of a sequenced genome?

In this study, two mosquito small RNA library data from *Aedes aegypti* were aligned to two closely related (*Anopheles gambiae* and *Drosophila melanogaster*) and one distantly related (*Bombyx mori*) insect genomes as proxy references to evaluate the accuracy of identification of known and novel miRNAs and their differential expression if the original genome sequence was not available. The outcomes of these analyses were validated by comparing the results when the original genome sequence was used as the standard reference genome. miRanalyzer and DSAP were selected for this study as they are user-friendly web servers with a short computational time and their overall approach towards miRNA detection has made them popular. The information in regards to the accuracy of miRNA discovery pipelines using closely related organisms’ genomes could provide valuable knowledge for scientists interested in the distribution patterns of miRNAs or discovering new miRNAs in non-model species.

## Results and Discussion

### Mapping Small Reads to Databases and Prediction of Conserved miRNAs

**Figure 1:**
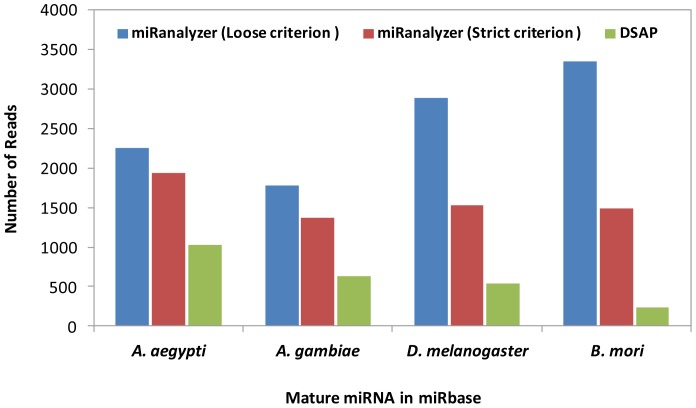
Number of reads from *A. aegypti* smRNA-seq data mapped to four selected species unique mature miRNA sequences in miRBase.

To identify known miRNAs in the libraries, we used the miRBase repository [23] which offers mature and precursor miRNA (pre-miRNA) sequences. As expected, more reads mapped to *A. aegypti*’s original mature miRNA set in comparison with those of other species in both analysis pipelines. More than 20% reduction was observed in the number of mapped reads using the strict criterion when other species miRNA sets were used as alignment proxy references (Fig. 1). The difference between the number of mapped reads when both mapping criteria were applied for the two closely related species, *A. aegypti* and *A. gambiae*, was around 20%, while applying the loose criterion (max 2 possible mismatches) led to significant enhancement in the number of reads mapped to known mature miRNAs in *D. melanogaster* and *B. mori* datasets (Fig. 1). In this case, allowance of two mismatches increased the coverage of data processing and showed the opposite pattern when the genome sequences were used as proxy alignment references.

The number of reads that were mapped to known mature miRNAs by DSAP was significantly less compared to miRanalyzer and this is due to inflexible aligning algorithm of DSAP (Fig. 1). The only reads that showed 100% identities were retained in DSAP, while miRanalyzer is able to analyse the isomers and potential variations in miRNA sequence. A software performance study showed DSAP and miRanalyzer keep the highest percentage of reads in the process of mapping among other miRNA discovery pipelines [24]. However, in the current analysis, the number of used reads demonstrated significant differences between the two pipelines. This indicates miRanalyzer utilized a larger portion of the available data for further analysis. miRanalyzer was developed as a sensitive learning algorithm to predict conserved and novel miRNAs with an AUC (Area Under Curve) of 97.9% and recall values of up to 75% on unseen data [25].

**Figure 2:**
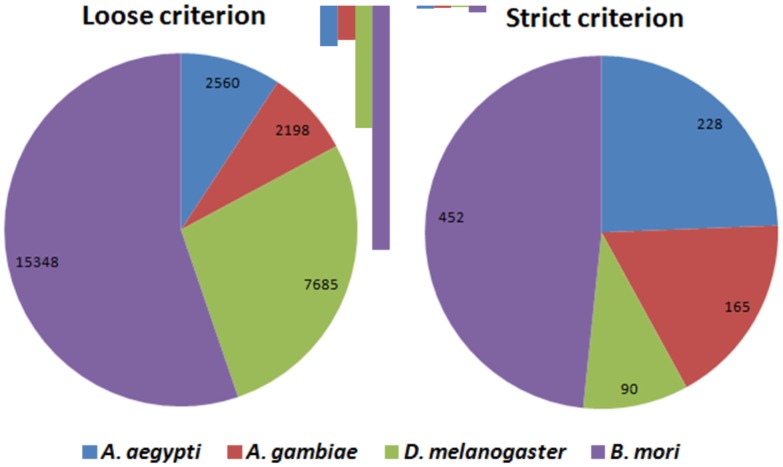
Number of reads from *A. aegypti* smRNA-seq data mapped to four selected species sequence of pre-miRNA hairpins in miRBase.

Figure 2 shows the number of reads mapped to known pre-miRNA sequences in miRBase, which in contrast to mature miRNA, using both mapping criteria, more reads were mapped to *B. mori* reference genome. Overall, the number of mapped reads when the strict criterion was applied, which allows just one mismatch in the reference dataset, was significantly lower than that of the loose criterion. Indeed, 83 and 13 times enrichment was observed in the number of mappable reads with the loose criterion in *D. melanogaster* and *A. aegypti,* respectively (Fig. 2). The pre-miRNA is more diverged than mature miRNA among different species and thus more mapped reads were expected with the loose criterion, which allows for more mismatches in the proxy reference genomes. Therefore, using loose criterion increased the coverage when a proxy reference was used and the results suggested that potentially there could be more homologous miRNAs in *A. aegypti* that have not been reported and remain to be discovered.

**Figure 3:**
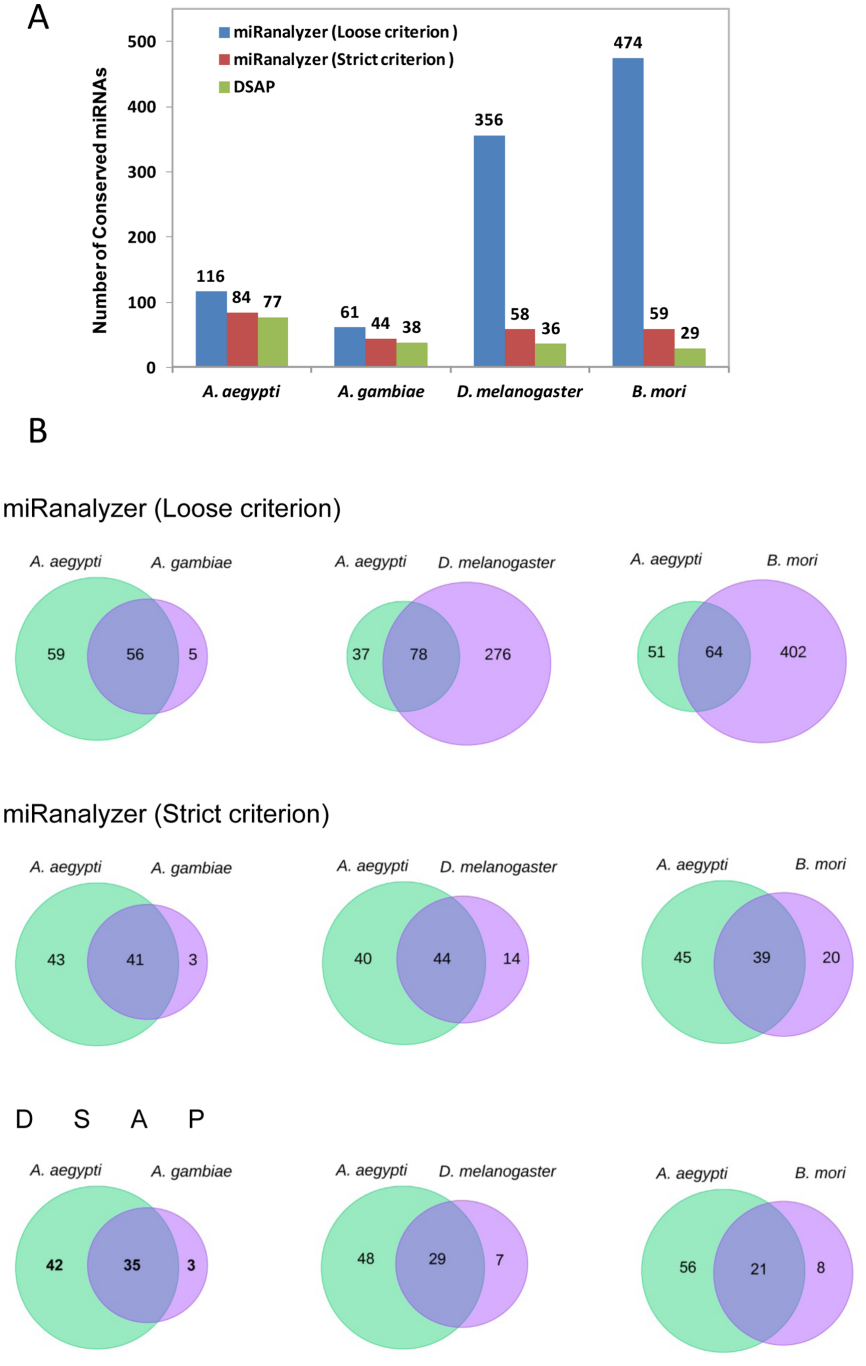
Number of conserved or known miRNAs identified in *A. aegypti* smRNA-seq data by miRanalyzer and DSAP. A) The total number of identified miRNAs with strict and loose criteria by mapping to species unique sequences in miRbase. B) Number of overlap or common miRNAs between *A. aegypti* and other selected species.

The two types of pipelines used in this study had the highest mature miRNA prediction success in software performance tests of *Homo sapiens* and *C. elegans* small RNA sequencing data [26]. In the current study, the number of predicted mature and pre-miRNAs were compared with species standard, which was defined as the total number of miRNAs for each species found in miRBase. When the reads were aligned to *A. aegypti*’s reported miRNAs using miRanalyzer, sequences of 84 known mature miRNAs were identified with strict criterion, which is almost 67.7% of the reported miRNAs from this species, while choosing the loose criterion increased this value to 93.5%, which is 116 miRNAs (Fig. 3A; Table 1).

**Table 1:** Identification rate of known mature and precursor miRNAs from *A. aegypti* in four selected species.

Species	loose criterion		strict criterion		DSAP (Mature)
	Mature	pre-miRNA	Mature	pre-miRNA	
*Aedes aegypti*	93.5%	84.2%	67.7%	30.7%	62.1%
*Anopheles gambiae*	93.8%	94.0%	65.7%	44.8%	58.5%
*Drosophila melanogaster*	83.8%	99.6%	13.6%	1.3%	8.4%
*Bombyx mori*	84.0%	97.3%	10.5%	1.4%	5.1%

In all cases, DSAP values were lower than those of miRanalyzer when strict criterion was applied. For example, only 5.1% of *B. mori* miRNA were identified by aligning *A. aegypti* small RNA reads to *B. mori* known miRNAs (Table 1). Using DSAP or strict criterion and other insect species data as proxy reference significantly reduced the number of detected miRNAs for *A. aegypti* small RNA library (Fig. 3A). Notably, using loose criterion dramatically increased the prediction values with *B. mori* and *D. melanogaster* proxy miRNA references (Fig. 3A). The results suggest that probably there are many insect conserved miRNAs which have not been reported from *A. aegypti* and their homologues could easily be identified in two genetically well annotated species *B. mori* and *D. melanogaster.* miRanalyzer detected 65.7% of *A. gambiae* mature miRNA sequences when *A. aegypti* small RNA reads were aligned to known *A. gambiae* miRNAs in miRBase, a value very close to its original species discovery rate (67.7% in *A. aegypti*). The data in Table 1 also show that the strict criterion provided around 10% of known miRNAs in phylogenetically distant species *D. melanogaster* and *B. mori,* suggesting high levels of diversification in miRNAs evolution. The majority of those miRNAs (around 84%) were identified with loose criterion in the two species, which shows that there are more than three mismatches or differences between *A. aegypti* mature sequences with those of two phylogenetically distant species. High level of conservation is expected between *D. melanogaster* and the two other mosquitoes (*Aedes* and *Anopheles*); however, previous comparative genomic analysis suggested that several fruit fly developmental genes could not be identified in mosquito genomes [27]. As a consequence, it is expected that a number of *Drosophila* miRNAs may not have orthologues in mosquitoes.

Research indicates that evolution of miRNAs is an ongoing process and the continuing innovation of novel miRNA families in different organisms is not the only way of evolution in this group of small RNAs but also the diversification of established families producing additional paralogues of miRNAs [28]. The pair-wise sequence identity of paralogous pre-miRNA sequences are often below 50–60% while their mature miRNA sequences show high level of conservation [29]. Previous studies have shown that the terminal loop is the least conserved part of pre-miRNAs [21], [30]. As shown in Table 1, with strict criterion, only less than 1.5% of pre-miRNAs were identified when *D. melanogaster* and *B. mori* genomes were used as proxy references.

The number of conserved miRNAs between *A. aegypti* and other selected species are presented in Fig. 3B. The results revealed that using the strict criterion 41, 44 and 39 miRNAs were common between *A. aegypti* and *A. gambiae*, *D. melanogaster* and *B. mori,* respectively. This overlap in detected miRNAs between species in each analysis suggests that several undescribed miRNAs potentially remain to be discovered in *A. aegypti*. The first *A. aegypti* miRNA repository was reported in 2009 by mapping 545 pyrosequencing data to the mosquito’s genome using the BLAST algorithm, which probably led to missing many potential miRNAs due to the allocated mapping criteria [31].

The results from this study also suggests high levels of conservation or similarity in miRNA repertoires among phylogenetically close species; for example 56 out of 61 *A. gambiae* miRNAs (∼92%) were identified with *A. aegypti* small RNA library while this value reduced to 13% (64 out of 474) in the phylogenetically distant species *B. mori* when loose criterion was applied (Fig. 3B). In addition, it has been reported that most mosquito miRNAs are conserved across divergent species with only 11 distinct miRNA genes to be mosquito-specific [31]–[33]. However, we only recalled around 50% of *A. aegypti* miRNAs when another mosquito’s genome (*A. gambiae*) or known miRNA dataset was used as proxy reference (Fig. 3B).

### Mapping Small RNAs to Genome References and Prediction of Novel miRNAs

**Figure 4:**
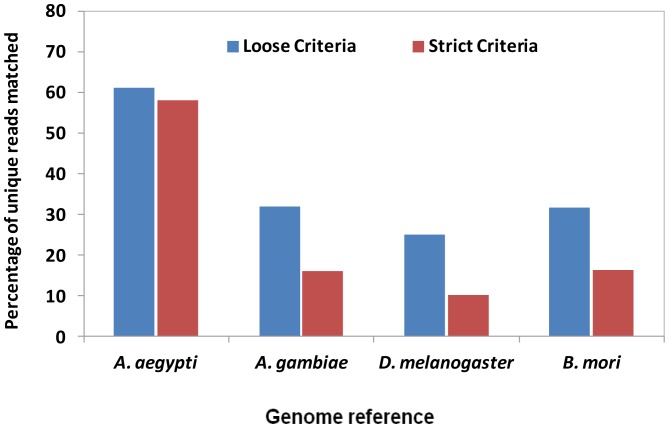
Percentage of unique reads from *A. aegypti* smRNA-seq data aligned to genome sequences from four different insects using strict or loose criterion.

As expected, the number of reads from the *A. aegypti* small RNA libraries that mapped to the *A. aegypti* genome sequence were significantly higher than matched reads to other proxy genome references. When the loose criterion was applied, the percentage of unique reads matched to the genome increased (Fig. 4). A laxer mapping to miRNAs or other libraries such as Rapbase, Rfam will remove more reads prior to the mapping to the genome. Therefore, less reads are mapped to the genome as they are removed at earlier stages. However, when other organism’s genomes were selected as proxy references, the number of non-matched reads considerably increased once the strict criterion was applied. The sensitivity of the Burrows–Wheeler Transform (BWT)-based algorithms such as Bowtie, which is used in miRanalyzer, decreases exponentially with the number of mismatches in genome reference. Improvements of mapping criteria are essential for the analysis of small RNA reads when proxy reference genomes are utilized for mapping purposes [34], [35]. DSAP is not able to perform this task because it is only designed for the identification of known miRNAs, which is independent of a complete genome sequence.

**Figure 5:**
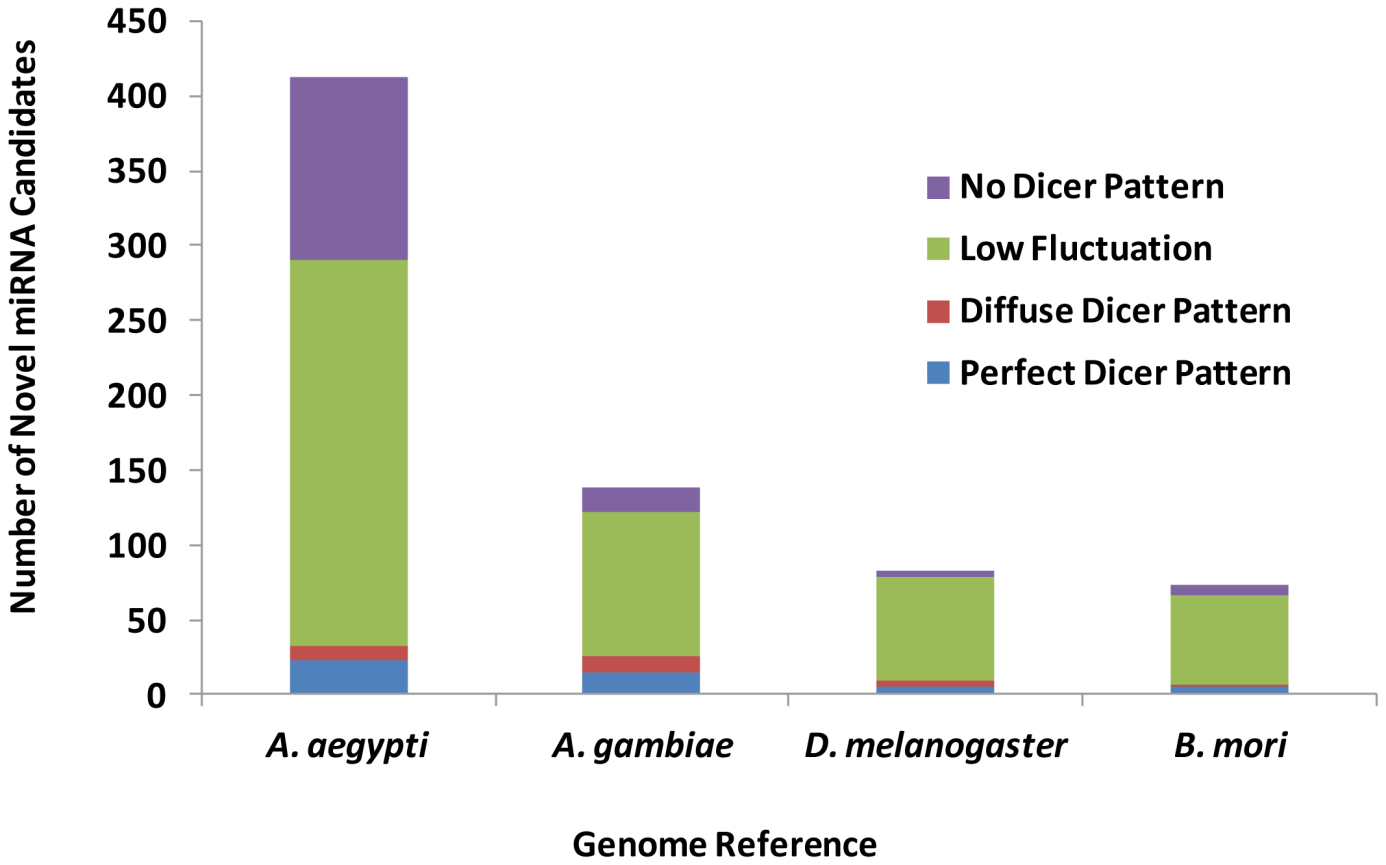
Number of novel miRNA candidates predicted in *A. aegypti* smRNA-seq data by mapping to four selected species genomes. *“Perfect Dicer pattern*: A perfect 3′ 2 nt overhang exists for the most expressed read and not more than 3 read clusters do exist on the pre-miRNA (one for the mature, one for the mature* and one for the loop sequences). *Diffuse Dicer patter*: A 3′ 1–4 nt overhang exists for the most expressed read and not more than 3 read clusters do exist on the pre-miRNA. *Low fluctuation*: No Dicer pattern has been detected but only one read cluster does exist. *No Dicer pattern*: No Dicer pattern has been detected or too many read cluster do exist” [25].

As mentioned above, identification of more potential novel miRNAs in *A. aegypti* was expected since in the previous step it was found that a high number of reads mapped to well-annotated miRNAs from *D. melanogaster* and *B. mori*. Accordingly, miRanalyzer detected more novel miRNAs in *A. aegypti* when the mosquito’s genome was used as reference, compared with when other organisms’ genomes were used as proxy references. These miRNAs were classified into four groups (Perfect Dicer pattern, Diffuse Dicer, low fluctuation and no Dicer patterns) based on the secondary structure of pre-miRNAs and the read alignments (Fig. 5). In all the four selected genomes, most of the novel miRNAs were categorized in low fluctuation and no Dicer patterns groups. Indeed, identification of novel miRNAs needs experimental validation but this *in silico* prediction confirmed that using the original species genome, the chance to identify new miRNAs is increased. Detection of fewer novel miRNAs, when small RNAs were mapped to *D. melanogaster* and *B. mori* genomes, is probably due to identification of many miRNAs that are already known in these species. In other words, some portions of miRNAs which were considered as novel (not reported) miRNAs in *A. aegypti* in this analysis have already been known or reported as homologues in other species.

Most of the novel miRNA candidates, which were identified by miRanalyzer, had very low copy numbers. Comparison of miRanalyzer with other similar web-based tools suggested that miRanalyzer is better suited to detect low-expressed novel miRNA candidates, and novel candidates represented by low abundant reads may not be excluded from library using its algorithm [24].

### Identification of Abundant miRNAs

The top twenty most abundant miRNAs in *A. aegypti* small RNA libraries were identified by miRanalyzer (Table 2) and DSAP (Table 3) through mapping reads to the four selected species references. Using loose or strict criteria did not make any significant differences in the profile of the most abundant miRNAs in *A. aegypti* compared with when its original genome was used as reference. miR-184, miR-275-3p, miR-317 were the most abundant miRNAs in all the different analyses. However, the important *A. aegypti’*s species-specific miRNA miR-2940 was missed when other species data were used as proxy references (Table 2 and 3).

**Table 2:** The top 20 most abundant *A. aegypti* miRNAs identified by miRanalyzer, when reads aligned to four species database.

*A. aegypti*		*A. gambiae*		*D. melanogaster*		*B. mori*	
strict	loose	strict	loose	strict	loose	strict	loose
miR-184	miR-184	miR-184	miR-184	miR-184-3p	miR-184-3p	miR-184-3p	miR-184-3p
miR-275-3p	miR-275-3p	miR-275	miR-275	miR-275-3p	miR-275-3p	miR-275-3p	miR-275-3p
miR-317	miR-317	miR-317	miR-317	miR-317-3p	miR-317-3p	miR-317-3p	miR-317-3p
miR-2940-3p+	miR-2940-3p+	–	–	–	–	–	–
miR-276-3p	miR-276-3p	miR-276-3p	miR-276-3p	miR-276a-3p	miR-276a-3p	miR-276-3p	miR-276-3p
miR-92b-3p	miR-92b-3p	miR-92b	miR-92b	miR-92b-3p	miR-92b-3p	–	miR-92b
miR-2940-5p+	miR-2940-5p+	–	–	–	–	–	–
miR-281-5p	miR-283	miR-283	miR-283	miR-281-2-5p	miR-281-2-5p	miR-281-5p	miR-281-5p
miR-283	miR-281-5p	miR-2	miR-2	miR-283-5p	miR-283-5p	miR-283-5p	miR-283-5p
miR-989	miR-989	miR-989	miR-989	miR-989-3p	miR-989-3p	miR-2a-3p	miR-2a-3p
miR-305-5p	miR-305-5p	miR-305	miR-305	miR-305-5p	miR-305-5p	miR-989a	miR-989a
miR-34-5p	miR-34-5p	miR-34	miR-34	miR-2a-3p	miR-2a-3p	miR-305-5p	miR-305-5p
miR-2a-3p	miR-2a-3p	–	–	miR-34-5p	miR-34-5p	miR-34-5p	miR-34-5p
miR-998	miR-998	–	–	miR-998-3p	miR-998-3p	miR-998	miR-998
miR-14	miR-14	miR-14	miR-14	miR-14-3p	miR-14-3p	miR-14-3p	miR-14-3p
miR-92a-3p	miR-92a-3p	miR-92a	miR-92a	miR-92a-3p	miR-92a-3p		miR-92a
miR-12-5p	miR-12-5p	miR-12	miR-12	miR-12-5p	miR-12-5p	miR-12	miR-12
miR-11-3p	miR-11-3p	miR-11	miR-11	miR-11-3p	miR-11-3p	miR-11-3p	miR-11-3p
miR-1889-5p+	miR-1889-5p+	–	–	–	miR-310-5p++	miR-2779++	miR-2779++
miR-306-5p	miR-263a-5p	miR-263a	miR-263a	miR-263a-5p	miR-263a-5p	miR-263a-5p	miR-306a-5p
		miR-988++	miR-306	miR-306-5p	miR-306-5p	miR-71-5p++	miR-263a-5p
		miR-970++	bantam++	miR-988-3p++	miR-304-5p++	bantam-3p++	miR-71-5p++
		bantam++	miR-988++	bantam-3p++		miR-970-3p++	
		miR-87++	miR-970++			miR-252-5p++	
		miR-8++	miR-87++				

*A. aegypti* species-specific miRNAs.

The miRNAs that appear at top 20 list when proxy references were used.

**Table 3:** The top 20 most abundant *A. aegypti* miRNAs identified by DSAP, when reads aligned to four species database.

*A. aegypti*	*A. gambiae*	*D. melanogaster*	*B. mori*
miR-184	miR-184	miR-184	miR-275
miR-275	miR-275	miR-275	miR-276-3p
miR-317	miR-276-3p	miR-276a	miR-281-5p
miR-2940-3p	miR-92b	miR-281-2-5p	miR-305
miR-276	miR-989	miR-305	miR-14
miR-92b	miR-305	miR-998	miR-34
miR-2940-5p	miR-34	miR-14	miR-263a
miR-989	miR-14	miR-12	miR-252
miR-305	miR-12	miR-11	miR-2a
miR-11-3p	miR-11	miR-970	miR-8*
miR-14	miR-92a	miR-988	miR-190
miR-12	miR-970	miR-252	miR-184
miR-34	miR-988	miR-34	miR-277
miR-11	miR-2	bantam	miR-7
miR-92a	miR-13b	miR-2a	miR-100
miR-1889-5p	miR-278	miR-13b	let-7
miR-71	miR-279	miR-278	miR-10
miR-263a	miR-190	miR-279	miR-1000
miR-970	miR-277	miR-9b	miR-11
bantam-3p	miR-7	miR-190	miR-279c

*A. aegypti* mature miRNA database as a reference showed high level of similarities between DSAP and miRanlayzer in the top 10 highly expressed miRNAs ranking. However, using other organisms’ known miRNA as reference made more variation in the list when DSAP was compared with miRanalyzer. The ranking profile produced by miRanalyzer is more reliable because of its flexible mapping algorithm and higher data coverage. For example, when *B. mori* was selected as a reference, miR-184, which is a very highly expressed miRNA in most cases, was not allocated in the top 10 by DSAP (Table 3).

In general, a significant proportion of miRNAs lack homologues among other species, which is likely due to species-specific adaptations. They are potentially the most interesting aspects of a species miRNA evolution, but they could simply be missed during annotation using inappropriate discovery pipelines and reference genomes. For example, *A. aegypti* species-specific miRNA aae-miR-2940 is one of the highly expressed miRNAs, which was only identified when *A. aegypti*’s genome was used as reference. The miRNA plays important roles in the maintenance of the endosymbiont *Wolbachia* in the mosquito and is involved in inhibition of replication of dengue virus in *Wolbachia*-infected *A. aegypti* [36], [37].

### Differentially Expressed miRNAs

**Figure 6:**
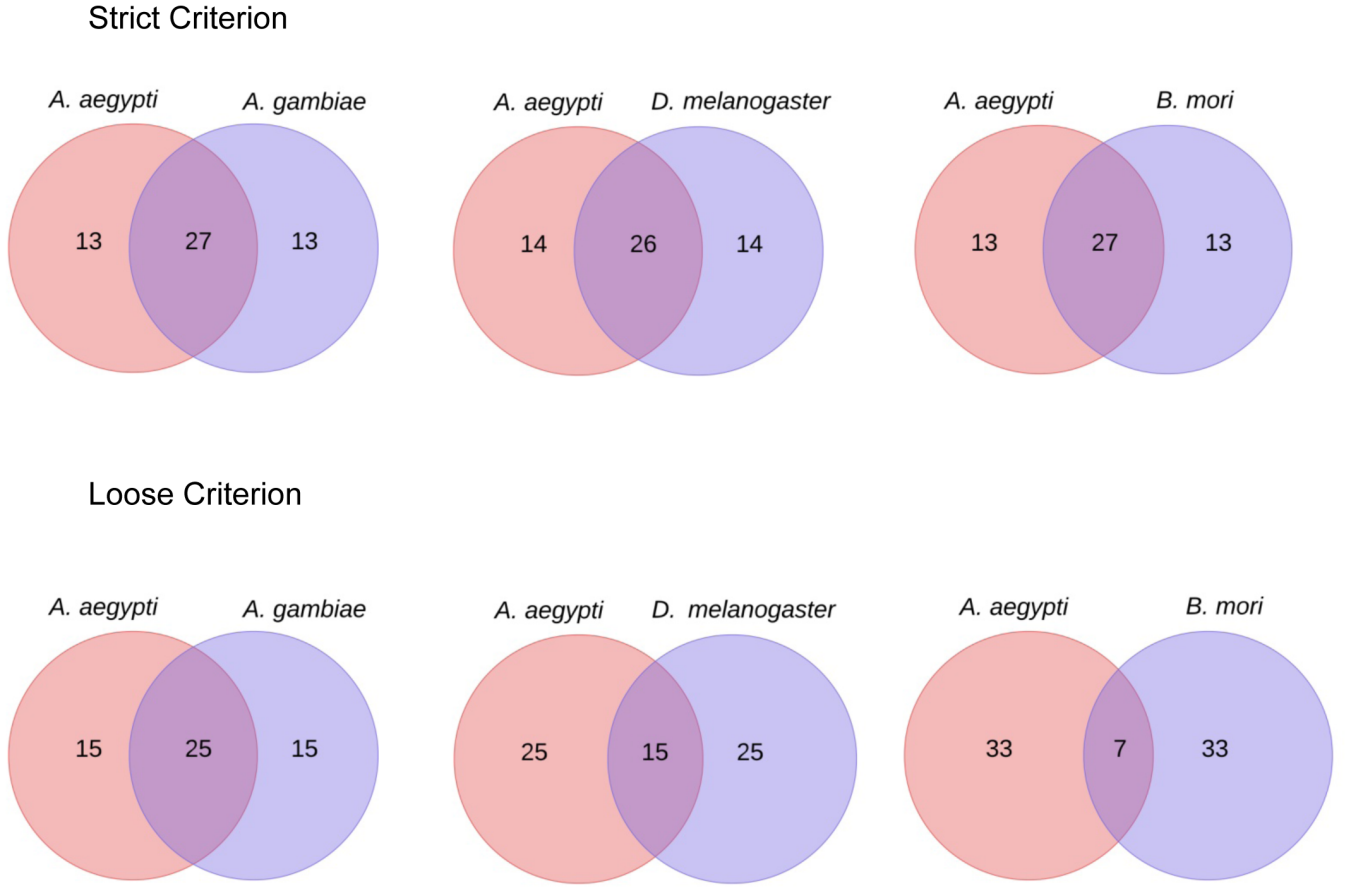
Unique highly differentially expressed miRNAs in Aag2 cell with and without *Wolbachia* infection when genomes of different selected insect species were used as references. Overlap areas show the number of common miRNAs in each comparison with high level of fold changes (more than 2).

In many instances, highly differentially expressed miRNAs under different treatments are of interest to researchers to find their biological functions in non-model species. To examine the impact of the species used as a genome reference to determine differentially expressed miRNA profiles, we used small RNA libraries from *A. aegypti Wolbachia*-infected and non-infected Aag2 cells for comparison [38]. miRNAs with an average read number of less than 10 were discarded from DESeq analysis output file and then the top 40 extremely up- and down-regulated miRNAs were selected for comparison (Fig. 6). When the strict criterion was used, around 27 out of 40 miRNAs were common between analyses when the genomes of *A. aegypti* and other species were used as references. In other words, using other organisms’ genomes as proxy references could provide about 67% of the outcome when the original (*A. aegypti*) genome was used as reference. However, this value was significantly reduced in phylogenetically distant species *B. mori* (17.7%) when loose criterion was applied (Fig. 6).

Differential expression values of 10 most abundant *A. aegypti* miRNAs in different analyses are presented in Table 4. Although in some cases the values are different in each analysis, the overall patterns are very similar. The results suggested that using other organisms’ genomes as reference did not change the most abundant miRNA differential expression profile which was calculated based on log 2 fold change. Although DSAP provides visualization interfaces for differential mature miRNA expression level, reporting non-normalized data is the main limitation of DSAP for this task. Due to this limitation, the value for log 2 fold changes in each analysis might vary with its corresponding value in miRanalyzer.

**Table 4:** Differential expression value of 10 most abundant *A. aegypti* miRNAs in different analyses when other insects data were used as reference.

miRNA	*A. aegypti*			*A. gambiae*			*D. melanogaster*			*B. mori*		
	Loose	Strict	DSAP	Loose	Strict	DSAP	Loose	Strict	DSAP	Loose	Strict	DSAP
miR-184	−0.5547	−0.5328	−0.42	−0.5355	−0.5343	−0.25	−0.3218	−0.5075	0.42	−0.3062	−0.5349	−1.77
miR-275-3p	−0.9623	−0.9398	−0.15	−0.9430	−0.9411	−0.75	−0.7293	−0.9143	0.75	−0.7131	−0.9411	−0.75
miR-317	−1.0387	−1.0176	−0.71	−1.0261	−1.0275	+∞	−0.7996	−0.9923	–	−0.7897	−1.0191	–
miR-2940-3p	0.2670	0.2888	0.56	–	–	–	–	–	–	–	–	–
miR-2940-5p	0.5286	0.5509	0.78	–	–	–	–	–	–	–	–	–
miR-276-3p	−0.4760	−0.4537	−0.11	−0.4568	−0.4551	−0.12	−0.2437	−0.4287	0.12	−0.2269	−0.4551	−0.12
miR-92b-3p	−0.7702	−0.7470	−∞	−0.7510	−0.7484	−0.41	−0.5393	−0.7172	–	−0.5189	–	–
miR-283	0.3487	0.3705	1.31	0.3712	0.3872	–	0.5826	0.4100	–	0.6011	0.3872	–
miR-281-5p	−1.2454	−1.2243	–	0.4644	0.6258	–	−1.0115	−1.1960	0.90	−0.9939	−1.2214	−0.90
miR-305-5p	−0.0195	0.0014	0.19	0.0000	0.0000	0.25	0.2137	0.0268	−0.25	0.2299	0.0000	0.25

Differential expression was calculated based on log 2 fold change.

## Conclusions

Analyzing small RNA-Seq data for miRNA discovery has classically required genomic sequences from the species of interest in order to map the reads to the reference genome. In the absence of genome sequences in many non-model organisms, a number of tools have been developed based on other species’ conserved miRNA datasets or other closely related species genome sequences as proxy references; however, the accuracy of these approaches have not been thoroughly evaluated; although there have been studies analysing the accuracy of quantitative RNA-Seq for gene expression in non-model species including *de novo* transcriptome assembly and the use of non-target species as reference scaffolds [39]. These studies have illustrated that using closely related species genome as reference incurs some losses in the number of predicted sequences and their expression data; in particular, the evolutionary distance between species introduces more biases and errors [39], [40].

Our findings indicated that the most abundant and conserved miRNAs can successfully be identified from a non-model species smRNA-Seq data by using closely related species genome references, but using a proxy genome reference does not lead to the identification of the whole miRNA profile in species without complete sequenced genome. In addition, this approach provides a robust starting place for the identification of differentially expressed miRNAs which are often of great interest to researchers when comparing samples from cells or organisms under different treatments (e.g. infected and non-infected). The overall pattern of differential expression for miRNAs with high copy numbers did not show any significant changes when other spehcies genomes were used as proxy references. Accordingly, around 67% of extremely up- or down-regulated miRNAs were identified by using strict criterion and other species genomes as proxy references. However, similar to transcriptome data studies, when the genome of phylogenetically distant species was used as reference, the number of identified differentially expressed miRNAs was reduced to around 13% when loose criterion was applied.

## Methods

### Dataset Preparation

Two small RNA libraries were generated from two *Aedes aegypti* (Diptera; Culicidae) Aag2 cell line samples using the Illumina Truseq™ Small RNA Preparation kit at LC Sciences Company (Houston, USA). The purified cDNA libraries were sequenced on Illumina GAIIx and raw sequencing reads (36 nts) were obtained using Illumina’s Sequencing Control Studio software version 2.8 followed by real-time sequencing image analysis and base-calling by Illumina's Real-Time Analysis version 1.8.70 (LC Sciences, Houston, USA). Two datasets with 1,409,306 and 3,347,907 raw reads were obtained from deep sequencing and a tab separated file with the read sequences and its counts were used as input file for miRanalyzer [41] and DSAP [18].

### miRNA Analysis Workflow

All reads with ‘N’ in their sequences and also those shorter than 17 bases or longer than 26 bases were removed from our datasets. To detect the number of known miRNAs, the filtered reads were aligned to the corresponding species miRNA sequences in miRBase and also they were mapped to the proxy genome references for predicting novel miRNAs.

An updated version of miRanalyzer, a web based server for the detection of known and prediction of novel miRNAs was used as the main pipeline for this analysis. This software is based on a random forest classifier and implements a highly accurate machine learning algorithm (Support Vector Machine) to predict new miRNA candidates from high throughput sequencing data [25]. The ultrafast short read aligner Bowtie was used to align the reads to the genomes and miRNA database (miRBase v. 19). DSAP, a deep-sequencing small RNA analysis pipeline was also used as control to increase our confident to exclude the impact of software performance on data analysis. DSAP takes a sequence tag file as input material and data processing is performed using Perl and Linux shell scripts [18]. For identification of known miRNAs the clustered reads were aligned with a non-redundant mature miRNAs reference, as default database, using word-match and Smith–Waterman algorithm [26].

Further, in a recent software performance evaluation study based on ROC curve (Receiver Operating Characteristic), an accuracy level of 68.3% and 67.3% were reported for miRanalyzer and DSAP, respectively [24]. This information increased the reliability of these tools for using in the current study.

The genomes of two closely related species *Anopheles gambiae* (Diptera: Culicidae) and *Drosophila melanogaster* (Diptera: Drosophilidae), and a distantly related species *Bombyx mori* (Lepidoptera; Bombycidae) were selected as mapping references to evaluate the accuracy of miRNA discovery pipeline based on other organisms genome sequence. *A. aegypti* genome sequence was used as control to measure the validity of the approach.

We implemented two sets of analyses based on loose and strict criteria with miRanalyzer, which were different in the number of mismatches in the genome and known miRNAs database. Strict criterion allowed a maximum of 1 mismatch in the genome, known miRNAs and homologous miRNAs, while loose criterion allowed a maximum of 2 mismatches in the genome and 3 mismatches for known and homologous miRNAs. For both criteria, 1 mismatch was allowed for other transcribed libraries such as Rfam and Rapbase. The software’s default seed alignment length for Bowtie (17 for Known miRNA, 19 for genomes and 20 for other transcribed libraries) was selected for all the analyses.

In DSAP analysis, hits with 100% sequence identity and full-length coverage with known miRNAs were considered as perfect BLAST hits and kept for further analysis. This software classified other sequence clusters, which showed low sequence homology with known miRNAs as putative novel miRNAs. However, we did not use this prediction due to lack of secondary structure information or any other complementary criteria for consideration as it is likely to produce a large number of false positives data [26].

Differential expression of miRNAs between two conditions was analyzed based on the DESeq package [42] on miRanalyzer server. DSAP is only able to calculate non-normalized miRNA expression levels between two samples using a log_2_-transformed colour matrix.

## References

[1] Ibanez-Ventoso C, Vora M, Driscoll M (2008) Sequence relationships among *C. elegans*, *D. melanogaster* and human microRNAs highlight the extensive conservation of microRNAs in biology. PLoS One 3: e2818. 18665242 10.1371/journal.pone.0002818 PMC2486268

[2] Bartel DP (2009) MicroRNAs: Target recognition and regulatory functions. Cell 136: 215–233. 19167326 10.1016/j.cell.2009.01.002 PMC3794896

[3] Fullaondo A, Lee SY (2012) Identification of putative miRNA involved in *Drosophila melanogaster* immune response. Dev Comp Immunol 36: 267–273. 21641929 10.1016/j.dci.2011.03.034

[4] Freitak D, Knorr E, Vogel H, Vilcinskas A (2012) Gender- and stressor-specific microRNA expression in *Tribolium castaneum* . Biol Lett 8: 860–863. 22628099 10.1098/rsbl.2012.0273 PMC3440968

[5] Asgari S (2013) MicroRNA functions in insects. Insect Biochemistry and Molecular Biology 43: 388–397. 23103375 10.1016/j.ibmb.2012.10.005

[6] Yu X, Zhou Q, Li S-C, Luo Q, Cai Y, et al (2008) The silkworm (*Bombyx mori*) microRNAs and their expressions in multiple developmental stages. PLoS One 3: e2997. 18714353 10.1371/journal.pone.0002997 PMC2500172

[7] Gomez-Orte E, Belles X (2009) MicroRNA-dependent metamorphosis in hemimetabolan insects. Proc Natl Acad Sci USA 106: 21678–21682. 19966227 10.1073/pnas.0907391106 PMC2799836

[8] Yu X, Zhou Q, Cai Y, Luo Q, Lin H, et al (2009) A discovery of novel microRNAs in the silkworm (*Bombyx mori*) genome. Genomics 94: 438–444. 19699294 10.1016/j.ygeno.2009.08.007

[9] Vasudevan S, Tong Y, Steitz JA (2007) Switching from repression to activation: MicroRNAs can up-regulate translation. Science 318: 1931–1934. 18048652 10.1126/science.1149460

[10] Hobert O (2007) miRNAs play a tune. Cell 131: 22–24. 17923083 10.1016/j.cell.2007.09.031

[11] Gu LQ, Wanunu M, Wang MX, McReynolds L, Wang Y (2012) Detection of miRNAs with a nanopore single-molecule counter. Expert Rev Mol Diagn 12: 573–584. 22845478 10.1586/erm.12.58 PMC3500609

[12] Van Roosbroeck K, Pollet J, Calin GA (2013) miRNAs and long noncoding RNAs as biomarkers in human diseases. Expert Rev Mol Diagn 13: 183–204. 23477558 10.1586/erm.12.134

[13] Chen X, Ba Y, Ma L, Cai X, Yin Y, et al (2008) Characterization of microRNAs in serum: a novel class of biomarkers for diagnosis of cancer and other diseases. Cell Res 18: 997–1006. 18766170 10.1038/cr.2008.282

[14] Wilbert ML, Yeo GW (2011) Genome-wide approaches in the study of microRNA biology. Wiley Interdiscip Rev Syst Biol Med 3: 491–512. 21197653 10.1002/wsbm.128 PMC3482411

[15] Lee RC, Ambros V (2001) An extensive class of small RNAs in *Caenorhabditis elegans* . Science 294: 862–864. 11679672 10.1126/science.1065329

[16] Belles X, Cristino AS, Tanaka ED, Rubio M, Piulachs MD (2012) Insect MicroRNAs: from molecular mechanisms to biological roles. In: Lawrence IG, editor. Insect Molecular Biology and Biochemistry. San Diego: Academic Press. 30–56.

[17] Wang W-C, Lin F-M, Chang W-C, Lin K-Y, Huang H-D, et al (2009) miRExpress: Analyzing high-throughput sequencing data for profiling microRNA expression. BMC Bioinformatics 10: 328. 19821977 10.1186/1471-2105-10-328 PMC2767369

[18] Huang P-J, Liu Y-C, Lee C-C, Lin W-C, Gan RR-C, et al (2010) DSAP: deep-sequencing small RNA analysis pipeline. Nucleic Acids Res 38: W385–W391. 20478825 10.1093/nar/gkq392 PMC2896168

[19] Wu W, Ren Q, Li C, Wang Y, Sang M, et al (2013) Characterization and comparative profiling of microRNAs in a sexual dimorphism insect, *Eupolyphaga sinensis* Walker. PLoS One 8: e0059016. 10.1371/journal.pone.0059016 PMC3631196 23620723

[20] Mehrabadi M, Hussain M, Asgari S (2013) MicroRNAome of *Spodoptera frugiperda* cells (Sf9) and its alteration following baculovirus infection. J Gen Virol 94: 1385–1397. 23407421 10.1099/vir.0.051060-0

[21] Etebari K, Hussain M, Asgari S (2013) Identification of microRNAs from *Plutella xylostella* larvae associated with parasitization by *Diadegma semiclausum.* . Insect Biochem Mol Biol 43: 309–318. 23352895 10.1016/j.ibmb.2013.01.004

[22] Yu D-B, Jiang B-C, Gong J, Dong F-L, Lu Y-L, et al (2013) Identification of novel and differentially expressed microRNAs in the ovaries of laying and non-laying ducks. J Integr Agric 12: 136–146.

[23] Kozomara A, Griffiths-Jones S (2011) miRBase: integrating microRNA annotation and deep-sequencing data. Nucleic Acids Res 39: D152–157. 21037258 10.1093/nar/gkq1027 PMC3013655

[24] Williamson V, Kim A, Xie B, McMichael GO, Gao Y, et al (2013) Detecting miRNAs in deep-sequencing data: a software performance comparison and evaluation. Brief Bioinform 14: 36–45. 23334922 10.1093/bib/bbs010 PMC3999373

[25] Hackenberg M, Sturm M, Langenberger D, Manuel Falcon-Perez J, Aransay AM (2009) miRanalyzer: a microRNA detection and analysis tool for next-generation sequencing experiments. Nucleic Acids Res 37: W68–W76. 19433510 10.1093/nar/gkp347 PMC2703919

[26] Li Y, Zhang Z, Liu F, Vongsangnak W, Jing Q, et al (2012) Performance comparison and evaluation of software tools for microRNA deep-sequencing data analysis. Nucleic Acids Res 40: 4298–4305. 22287634 10.1093/nar/gks043 PMC3378883

[27] Behura SK, Haugen M, Flannery E, Sarro J, Tessier CR, et al (2011) Comparative genomic analysis of *Drosophila melanogaster* and vector mosquito developmental genes. Plos One 6: e21504. 21754989 10.1371/journal.pone.0021504 PMC3130749

[28] Hertel J, Lindemeyer M, Missal K, Fried C, Tanzer A, et al (2006) The expansion of the metazoan microRNA repertoire. BMC Genomics 7: 25. 16480513 10.1186/1471-2164-7-25 PMC1388199

[29] Tanzer A, Stadler PF (2006) Evolution of MicroRNAs. In: Ying SY, editor. Methods in Molecular Biology: Humana Press Inc, 999 Riverview Dr, Ste 208, Totowa, Nj 07512-1165 USA. 335–350. 10.1385/1-59745-123-1:335 16957387

[30] Li SC, Chan WC, Hu LY, Lai CH, Hsu CN, et al (2010) Identification of homologous microRNAs in 56 animal genomes. Genomics 96: 1–9. 20347954 10.1016/j.ygeno.2010.03.009

[31] Li S, Mead EA, Liang S, Tu Z (2009) Direct sequencing and expression analysis of a large number of miRNAs in *Aedes aegypti* and a multi-species survey of novel mosquito miRNAs. BMC Genomics 10: 581. 19961592 10.1186/1471-2164-10-581 PMC2797818

[32] Skalsky RL, Vanlandingham DL, Scholle F, Higgs S, Cullen BR (2010) Identification of microRNAs expressed in two mosquito vectors, *Aedes albopictus* and *Culex quinquefasciatus* . BMC Genomics 11: 119. 20167119 10.1186/1471-2164-11-119 PMC2834634

[33] Gu J, Hu W, Wu J, Zheng P, Chen M, et al (2013) miRNA genes of an invasive vector mosquito, *Aedes albopictus* . PLoS One 8: e67638. 23840875 10.1371/journal.pone.0067638 PMC3698096

[34] Zhang G, Fedyunin I, Kirchner S, Xiao C, Valleriani A, et al (2012) FANSe: an accurate algorithm for quantitative mapping of large scale sequencing reads. Nucleic Acids Res 40: e83. 22379138 10.1093/nar/gks196 PMC3367211

[35] Lunter G, Goodson M (2011) Stampy: A statistical algorithm for sensitive and fast mapping of Illumina sequence reads. Genome Res 21: 936–939. 20980556 10.1101/gr.111120.110 PMC3106326

[36] Hussain M, Frentiu FD, Moreira LA, O'Neill SL, Asgari S (2011) *Wolbachia* uses host microRNAs to manipulate host gene expression and facilitate colonization of the dengue vector *Aedes aegypti* . Proc Natl Acad Sci USA 108: 9250–9255. 21576469 10.1073/pnas.1105469108 PMC3107320

[37] Zhang G, Hussain M, O'Neill SL, Asgari S (2013) *Wolbachia* uses a host microRNA to regulate transcripts of a methyltransferase, contributing to dengue virus inhibition in *Aedes aegypti* . Proc Natl Acad Sci USA 110: 10276–10281. 23733960 10.1073/pnas.1303603110 PMC3690878

[38] Frentiu FD, Robinson J, Young PR, McGraw EA, O'Neill SL (2010) *Wolbachia*-mediated resistance to Dengue virus infection and death at the cellular level. PLoS One 5: e13398. 20976219 10.1371/journal.pone.0013398 PMC2955527

[39] Hornett EA, Wheat CW (2012) Quantitative RNA-Seq analysis in non-model species: assessing transcriptome assemblies as a scaffold and the utility of evolutionary divergent genomic reference species. BMC Genomics 13: 361. 22853326 10.1186/1471-2164-13-361 PMC3469347

[40] Gibbons JG, Janson EM, Hittinger CT, Johnston M, Abbot P, et al (2009) Benchmarking next-generation transcriptome sequencing for functional and evolutionary genomics. Mol Biol Evol 26: 2731–2744. 19706727 10.1093/molbev/msp188

[41] Hackenberg M, Rodriguez-Ezpeleta N, Aransay AM (2011) miRanalyzer: an update on the detection and analysis of microRNAs in high-throughput sequencing experiments. Nucleic Acids Res 39: W132–W138. 21515631 10.1093/nar/gkr247 PMC3125730

[42] Anders S, Huber W (2010) Differential expression analysis for sequence count data. Genome Biol 11: R106. 20979621 10.1186/gb-2010-11-10-r106 PMC3218662

